# A network-based approach to identify substrate classes of bacterial glycosyltransferases

**DOI:** 10.1186/1471-2164-15-349

**Published:** 2014-05-08

**Authors:** Aminael Sánchez-Rodríguez, Hanne LP Tytgat, Joris Winderickx, Jos Vanderleyden, Sarah Lebeer, Kathleen Marchal

**Affiliations:** Department of Microbial and Molecular Systems, KU Leuven, Centre of Microbial and Plant Genetics, Kasteelpark Arenberg 20, box 2460, Leuven, B-3001 Belgium; Department of Bioscience Engineering, University of Antwerp, Groenenborgerlaan 171, Antwerp, B-2020 Belgium; Department of Biology, Functional Biology, KU Leuven, Kasteelpark Arenberg 31, box 2433, Leuven, B-3001 Belgium; Department of Plant Biotechnology and Bioinformatics, Ghent University, Technologiepark 927, Ghent, B-9052 Belgium; Department of Information Technology, Ghent University, IMinds, Gent, 9052 Belgium; Departamento de Ciencias Naturales, Universidad Técnica Particular de Loja, San Cayetano Alto s/n, Loja, Ecuador

**Keywords:** Network-based prediction, Sequence-based prediction, Bacterial glycosylation, Glycosyltransferases, *Lactobacillus rhamnosus GG*, *Campylobacter jejuni*

## Abstract

**Background:**

Bacterial interactions with the environment- and/or host largely depend on the bacterial glycome. The specificities of a bacterial glycome are largely determined by glycosyltransferases (GTs), the enzymes involved in transferring sugar moieties from an activated donor to a specific substrate. Of these GTs their coding regions, but mainly also their substrate specificity are still largely unannotated as most sequence-based annotation flows suffer from the lack of characterized sequence motifs that can aid in the prediction of the substrate specificity.

**Results:**

In this work, we developed an analysis flow that uses sequence-based strategies to predict novel GTs, but also exploits a network-based approach to infer the putative substrate classes of these predicted GTs. Our analysis flow was benchmarked with the well-documented GT-repertoire of *Campylobacter jejuni* NCTC 11168 and applied to the probiotic model *Lactobacillus rhamnosus* GG to expand our insights in the glycosylation potential of this bacterium. In *L. rhamnosus* GG we could predict 48 GTs of which eight were not previously reported. For at least 20 of these GTs a substrate relation was inferred.

**Conclusions:**

We confirmed through experimental validation our prediction of WelI acting upstream of WelE in the biosynthesis of exopolysaccharides. We further hypothesize to have identified in *L. rhamnosus* GG the yet undiscovered genes involved in the biosynthesis of glucose-rich glycans and novel GTs involved in the glycosylation of proteins. Interestingly, we also predict GTs with well-known functions in peptidoglycan synthesis to also play a role in protein glycosylation.

**Electronic supplementary material:**

The online version of this article (doi:10.1186/1471-2164-15-349) contains supplementary material, which is available to authorized users.

## Background

The glycome, playing a crucial role in allowing bacteria to establish environment- and host-specific interactions [1, 2] consists of a wide variety of glycoconjugates, i.e. glycans being covalently linked to other macromolecules. In Gram-negatives, these glycoconjugates occur mainly in the outer membrane as a thin layer of peptidoglycan (PG) and lipopolysaccharides (LPS) or lipo-oligosaccharides (LOS). Across the outer membrane, exopolysaccharides (EPS) or capsular polysaccharides (CPS), glycoproteins and glycolipids can further decorate the cell surface [2]. In Gram-positives, which in contrast to Gram-negatives lack an outer membrane, complex polymers such as teichoic acids in *Firmicutes* and lipoglycans in *Actinobacteria* strengthen a thick layer of PG. CPS or EPS are also often found as most external layer in Gram-positive bacteria. Bacteria can also produce intracellular glycoconjugates, such as glycosylated secondary metabolites and storage polysaccharides like glycogen [2].

Glycosyltransferases (GTs), transferring sugar moieties from an activated donor to a specific substrate [3], are key enzymes in the biosynthesis of glycoconjugates. Depending on their specificity, the substrates of GTs range from lipids, proteins, saccharides, nucleic acids to small molecules [3]. In bacteria, two different glycosylation mechanisms have been described: sequential glycosylation, in which either soluble or membrane-associated GTs transfer glycan monomers directly to the final substrate and *en bloc* glycosylation, in which the sugar moiety is first assembled and only then transferred to the final substrate by an specialized GT (oligosaccharyltransferase (OST) or polymerase) [4, 5]. The latter mechanism is by far the best documented, and is involved in the biosynthesis of heteropolymeric EPS/CPS, O-antigens in LPS, and even PG biosynthesis, highlighting the commonalities in the biosynthesis of these glycoconjugates [5]. Apart from their general role in glycosylation, the specificities of most of the GTs and the cellular role of their end products are still largely unknown. In addition, most of the substrate specificities of GTs involved in LPS, PG and glycoproteins have been described in Gram-negatives [6, 7]*,* while glycosylation in Gram-positives is much less studied.

Whereas sequence-based predictions have shown useful to identify potential GTs [8–10], predicting the specificity of those identified GTs is less trivial, definitely for prokaryotes for which no clear sequence motifs determining substrate specificity have been described [11]. In addition, many GTs and OSTs show substrate promiscuity [12, 13], hampering the identification of clear substrate motifs.

To improve the annotation of GTs in prokaryotes, we developed an analysis flow that uses a sequence-based strategy to predict GTs and a network-based approach [14] to identify links between these predicted GTs and other genes/proteins. Although such links do not give insights into the precise biochemical mechanisms of a GT with its substrate, they aid in relating the GT to possible classes of molecules that could accept the sugar moieties from these GTs (referred to as substrate classes).

We tested our analysis flow on the genome of *C. jejuni* NCTC 11168, in which the important classes of glycoconjugates (*N-* and *O-*glycoproteins, PG, LOS, and CPS) are well characterized [4].

Further applying our analysis flow on the probiotic bacterium *Lactobacillus rhamnosus* GG provided a comprehensive re-annotation of putative GTs in this species, the possible substrate classes of these GTs and their mode of action. These predictions are a very useful resource for experimentalists, predominantly because the study of (protein) glycosylation in lactobacilli and related organisms is not straightforward [15]. Our predictions unveil putative novel mechanisms of (protein) glycosylation, involving the potential, promiscuous role of GTs with known function in PG biosynthesis.

## Methods

### Bacterial proteomes

The proteomes and current genome annotations of *Lactobacillus rhamnosus* GG (NC_013198.1) and *Campylobacter jejuni* NCTC 11168 (NC_002163.1) were obtained from GenBank (http://www.ncbi.nlm.nih.gov/genbank/).

### Hidden Markov Model profile searches

Hidden Markov Models (HMMs) describing known GT signatures were collected from SUPERFAMILY (http://supfam.cs.bris.ac.uk/SUPERFAMILY/), CAZy (http://www.cazy.org/) and Pfam (http://pfam.sanger.ac.uk/) and subdivided into three groups depending on their expected specificity for GTs (Table 1). For CAZy, a thorough search of this database was performed, and all the HMMs covering GT classes that had bacterial representatives were included in our analysis (see below).

**Table 1:** **Summary of the Hidden Markov Models (HMMs) used to screen for glycosyltransferases in the proteomes of** ***Campylobacter jejuni*** **NCTC 11168 and** ***Lactobacillus rhamnosus*** **GG**

HMM group	Description	Database	Reference
I	Rossmann-fold domains	SUPERFAMILY	Ha *et al*., 2001 [16]
			Egelund *et al*., 2004 [10]
			Lairson *et al.,* 2008 [3]
			Hansen *et al*., 2010 [8]
II	Sugar transferase	SUPERFAMILY	Egelund *et al*., 2004 [10]
			Hansen *et al*., 2010 [8]
	UDP-Glycosyltransferase	SUPERFAMILY	Egelund *et al*., 2004 [10]
			Hansen *et al*., 2010 [8]
III	Transglycosylase (PF00912)	Pfam/CAZy	Di Guilmi *et al*., 2003 [17]
	Glycosyltransferase WecB/TagA/CpsF (PF03808)	Pfam/CAZy	Maldonado-Barragán *et al.,* 2011 [18]
	Bacterial sugar transferase (PF02397)	Pfam	Yoshida *et al.,* 1998 [19]
			Provencher *et al*., 2003 [21]
	Oligosaccharyltransferase STT3 subunit (PF02516)	Pfam/CAZy	Baïet *et al*., 2011 [22]
	DAD family (PF02109)	Pfam	Silberstein *et al*., 1995 [24]
	OST3/OST6 family (PF04756)	Pfam/CAZy	Knauer *et al*., 1999 [23]
	Glycosyltransferase family 25 (PF01755)	Pfam/CAZy	Campbell *et al*., 1997 [25]
	Glycosyltransferase family 28 (PF04101)	Pfam/CAZy	Mengin-Lecreulx *et al*., 1991 [26]
	Glycosyltransferase family 9 (PF01075)	Pfam/CAZy	Campbell et al., 1997 [25]

**HMM group**: HMMs were grouped according to their expected specificity for glycosyltransferase activity in an increasing order. **Description**: description of the HMM. The Pfam model id is also provided. **Database**: source of the model. **Reference**: bibliographic citation supporting the inclusion of the corresponding HMM in the analysis.

The first and least specific group contains the HMM representing ‘Rossmann-fold domains’, which are known to resemble the GT-A and GT-B folds typical for GTs using sugar nucleotides as donor [3, 8, 16]. A second group comprises the HMMs for ‘Sugar transferases’ and ‘UDP-Glycosyltransferases’ respectively, both HMMs of intermediate specificity covering a broad class of GTs [8, 10]. A last group combines a set of more GT-specific HMMs (10 in total), all of which are based on a small number of family-specific sequences [17–26]. This group combines HMMs extracted from CAZy [27], representative for enzymes that catalyze glycosidic bonds (strictu-sensu GTs) with HMMs extracted from Pfam [28] that are representative for non-Leloir GTs that use non-nucleotide sugar donors or oligo/polysaccharides. Enzymes involved in the transfer of the sugar moiety to the final substrate (such as OTases and priming GTs) are examples of this latter class of non-Leloir GTs.

The collected HMMs were used to screen entire proteomes (*C. jejuni* NCTC 11168 and *Lactobacillus rhamnosus* GG) with *hmmsearch* from the HMMER package version 2.2 [29]. Hits were filtered using an E-value cut-off of 0.1.

### Protein fold recognition

The profile based fold recognition method pGenTHREADER [30], accessible via the PSIRED server (http://bioinf.cs.ucl.ac.uk/psipred/) was used to detect known GT-A/GT-B folds in proteins predicted to be GTs by the HMM search. Each of the input sequences was aligned against a library of 3D folds based on CATH v3.3 (the Protein Structure Database, available at http://www.cathdb.info/) by pGenTHREADER. The library of 3D folds contains a total of 684 PDB structures of known GTs. Putative GTs were only retained if they predicted fold showed significant homology (net score > 46) to the one of a resolved 3D structure with known GT activity present in the library (refined set). We selected a cutoff > 46 on the net score of pGenTHREADER since any values higher than this threshold are categorized as HIGH to CERTIFIED confidence predictions (default conservative setting of the tool).

### Detecting functional partners of glycosyltransferases

The STRING database (http://string-db.org/) was used as the source of functional networks [14, 31]. We interrogated STRING using as queries our predicted GTs from both *L. rhamnosus* GG and *C. jejuni* NCTC 11168 to retrieve the network of functional partners associated to each query (query-based subnetwork). We only considered functional interactions with a score higher than 0.7, which is the default value in STRING for high confidence interactions. A total of 1112 functional interactions were retrieved for *L. rhamnosus* GG, supported by 2338 independent evidences distributed as follows: 1682 evidences based on the genomic context of the interacting partners (e.g. physical closeness, co-occurrence in closely related species, gene fusion events); 153 evidences based on the co-expression of the interacting partners; 28 evidences derived from high-throughput experiments (e.g. protein-protein interaction data); 465 evidences derived from the literature (text-mining). For *C. jejuni* NCTC 11168 a total of 1727 functional interactions were retrieved supported by 3190 independent evidences from the following data sources: 2520 evidences based on the genomic context of the interacting partners; 47 evidences based on co-expression; 37 evidences from high-throughput experiments; 584 evidences derived from the literature.

Gene Ontology annotation files for *L. rhamnosus* GG and *C. jejuni* NCTC 11168 were obtained from http://www.ebi.ac.uk/GOA/proteomes.html. To calculate which functional GO classes were enriched amongst interacting partners of a certain GT, we used the hypergeometric test, corrected for multiple testing using False Discovery Rate [32].

We then created ‘consensus networks’ that combine the local network neighborhood of all GTs, predicted to belong to the same specificity class and of which the local subnetworks are enriched in the same GO terms. GT-specific subnetworks were merged in a consensus network by retaining the edges from all the composing subnetworks that either reflect GT-GT interactions, interactions between a GT and one or more transmembrane proteins (membrane associations) or interactions between GTs and proteins with predicted glycosylation signals (predicted protein substrate relation).

### Detection of putative protein glycosylation sites

Glycosylation sites were predicted in the proteomes of *C. jejuni* NCTC 11168 and *L. rhamnosus* GG using the GlycoPP webserver (http://www.imtech.res.in/raghava/glycopp/), specially developed for the analysis of prokaryotic protein sequences. Predictions were made using the hybrid approaches: BPP + ASA (for N-glycosites predictions) and PPP + ASA (for *O*-glycosites prediction) as suggested by the developers. A SVM threshold of 0.5 was used to reduce the probability of false positive predictions.

### Prediction of transmembrane helices

Transmembrane helices were predicted using the TMHMM server version 2.0 (http://www.cbs.dtu.dk/services/TMHMM/).

### Benchmark

The available data on glycosylation in the paradigm organism *C. jejuni* NCTC 11168 was used for benchmarking purposes and helped us to fine-tune and evaluate our workflow. *C. jejuni* is considered as a model for bacterial glycosylation, since it can not only *N-* and *O*- glycosylate proteins by both sequential and *en bloc* transfer [33, 34], but also produces a wide variety of glycoconjugates, including PG, LOS and CPS. Because glycosylation is extensively studied in *C. jejuni* NCTC 11168 we used this model system to compile a literature benchmark dataset. We obtained information on 10 proteins with experimentally verified glycosyltransferase activity and known substrate specificity in *C. jejuni* (Cj1124c, Cj1125c, Cj1126c, Cj1127c, Cj1128c and Cj1129c involved in protein N-glycosylation and Cj1133, Cj1136, Cj1139c and Cj1148 involved in LOS biosynthesis). Proteins annotated in *C. jejuni* NCTC 11168 as GTs based on indirect evidence (e.g. through homology assignment) were omitted from the benchmark dataset.

### Reannotation of GTs in *C. jejuni* and *L. rhamnosus GG* based on our predictions and literature

For the GTs that were previously annotated with a GT-related function, a simplified annotation is proposed when the evidence on the exact GT activity is not available for *L. rhamnosus* GG (such as for *LGG_00279, LGG_00280* and *LGG_00281*). In addition, gene names inferred from non-strong homology searches (i.e. BLASTn E-value > 0.01) were removed (e.g. *LGG_00348*). For GTs putatively involved in polysaccharide biosynthesis (*LGG_00279-LGG_00283*, see below), gene names were corrected in agreement with the correct gene nomenclature [35].

### Experimental work

*L. rhamnosus* GG and its mutant derivatives were grown in MRS without agitation. A new Δ*welI*::Tc^R^ gene deletion mutant, lacking the *LGG_02047* gene, termed CMPG 10811, was constructed as described earlier [36], using the pro-7946 (5′-ATACTAGTTCTTATCATAGTTTCCAGACC-3′) and pro-7947 (5′-ATCCCGGGGTGGGGAACTTGCTG-3′) primers. As this is a gene deletion mutant in an operon, polar effects can not completely be ruled out. Total EPS determination, monomer analysis and adhesion assays were performed as previously described [37]. Statistical analysis (One-way ANOVA) was performed using GraphPad Prism 6 on data corresponding to three technical repeats of three independent biological samples.

## Results

### Annotating putative glycosyltransferases

**Figure 1:**
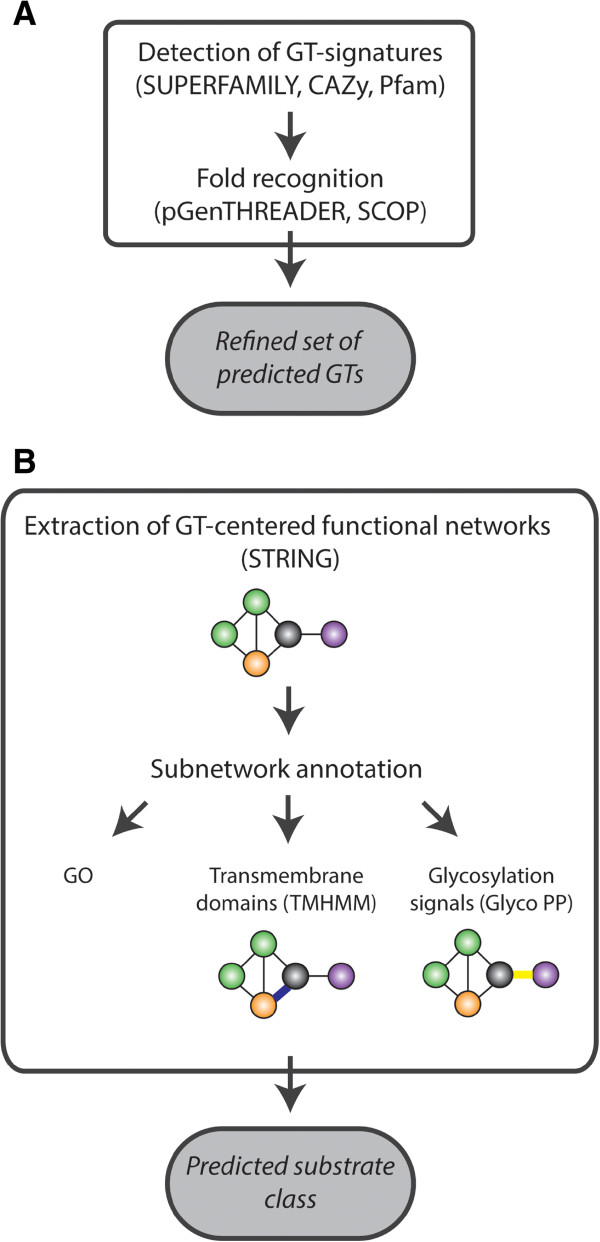
**Glycosyltransferase annotation flow. A**: Genome-wide annotation of glycosyltransferases (GTs). Glycosyltransferases are predicted by scanning the proteomes of the studied species for GT-specific signatures using Hidden Markov Models (HMM) from SUPERFAMILY, CAZy and Pfam. An additional fold recognition filtering step is applied to only retain those genes containing a three-dimensional fold (inferred by the PGenTHREADER algorithm) with significant homology to folds present in experimentally confirmed GTs (deposited in the SCOP database). **B**: Predicting GT substrate class and putative mode of action (bottom panel). The local network neighborhood of each query GT (black node) in a functional interaction network (STRING) is used to extract a GT-specific local subnetwork for each query GT. The local subnetwork of a GT comprises predicted functional partners (proteins being functionally related to the query GT). Based on the GO enrichment analysis of these genes in this local subnetwork, the substrate class of the query GT is derived. To gain information on the mode of glycosylation, the GT specific local subnetwork is further annotated with either membrane associations between a query GT and a predicted transmembrane protein (blue edge) and with relations indicative for protein glycosylation (yellow edge).

To predict additional GTs, we used an HMM based screening (Figure 1A). To maximize the sensitivity of our screening, the heterogeneous functional family of GTs was represented by a collection of 12 different HMMs, each of which captures a different characteristic of known GTs (Table 1). These 12 HMMs were subdivided into three groups depending on their expected specificity for GTs, referred to as respectively I) ‘Rossmann-fold domains’, II) ‘Sugar transferase’ and ‘UDP-Glycosyltranferase’ and III) a set of nine more GT-specific HMMs.

As HMM-based screenings, definitely those performed with the least GT-specific HMMs, tend to also find many non-specific hits (false positives), predictions were further filtered using a protein fold recognition step: GTs predicted by the HMM profiling were only retained if they contained a three-dimensional fold with significant homology to folds present in experimentally confirmed GTs from any species (referred to as the refined set in Figure 1) (see Methods).

**Figure 2:**
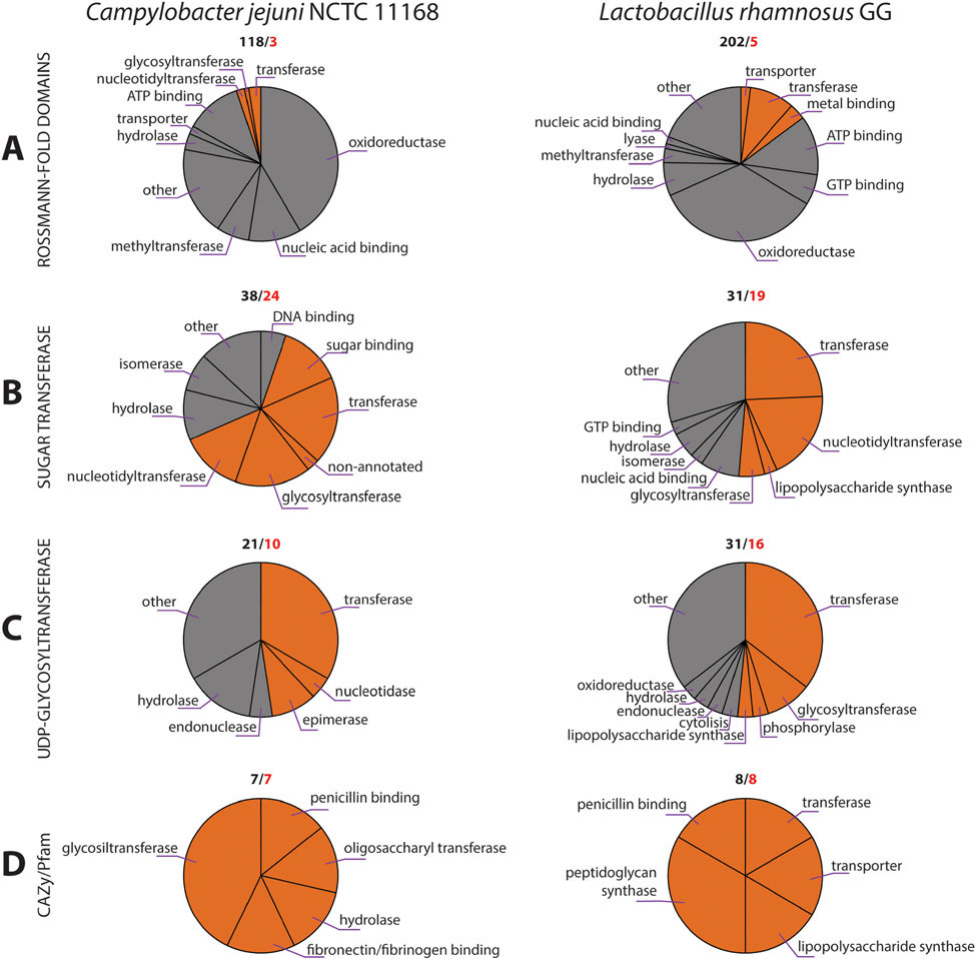
**Annotated glycosyltransferases.** Results for the model system *Campylobacter jejuni* are shown on the left panel and for *L. rhamnosus* GG on the right panel. Putative GTs were predicted using an HMM based screening. **A**: results obtained with an HMM recognizing ‘Rossmann-fold domains’, expected to be the HMM with the lowest specificity towards GTs (Table 1, class I). **B** and **C**: results obtained with a family of HMMs of intermediate specificity for GTs (Table 1, class II). **D**: results obtained with the class of HMMs, most specific for GTs (Table 1, class III). Pie charts indicate the extent to which different functional classes were enriched amongst the predictions obtained with the respective classes of HMMs. Slices indicated in red on the pie chart correspond to the functional classes of the predictions that were retained after the fold recognition filtering step. For each group of HMMs, the total number of predictions is denoted in black on top of every pie chart and the number of predictions retained after applying the fold recognition step is denoted in red.

The results of the HMM based screening in both *L. rhamnosus* GG and *C. jejuni* NCTC 11168 before and after filtering with the fold based predictions are shown in Figure 2, together with the most abundant GO categories present amongst the predicted GTs. Filtering successfully reduced potential false positive predictions, for instance, a large fraction of oxidoreductases (all binding the cofactor NAD) obtained by screening with the least specific ‘Rossmann-fold domain’ HMM were removed after the fold recognition based filtering (Figure 2A). The three predictions in *C. jejuni* (Additional file 1: Table S1) and the five in *L. rhamnosus* GG (Table 2) made by the ‘Rossmann-fold domain’ HMM and retained after the fold recognition could not be retrieved by any of the other HMM models, showing the added value of also using this least specific class of HMMs. Screening with the ‘Sugar transferases’ and ‘UDP-glycosyltransferases’ HMMs in contrast resulted in predictions that were quite GT-specific, as indeed approximately 50% of the originally obtained predictions also contain a GT-like fold (Figure 2B and C). Fold-based filtering here removed mainly predicted DNA-binding proteins, as their mechanism of binding DNA is also based on recognizing the sugar moieties of the nucleotides. As expected, screening with the HMMs obtained from Pfam and CAZy resulted both in *C. jejuni* NCTC 11168 and *L. rhamnosus* GG in the highest fraction of hits that also displayed a GT-like fold (Figure 2D).

**Table 2:** **Updated annotation of glycosyltransferases predicted in the genome of** ***Lactobacillus rhamnosus*** **GG**

Locus tag	Current annotation	Proposed annotation	HMM	Evidence	Reference
LGG_00279	*welA*; dTDP-rhamnosyl transferase *rfbF*	*wclA;* glycosyltransferase (putative cell wall polysaccharide biosynthesis)	Sugar transferase	Conservation	Kankainen *et al.,* 2009 [44]
LGG_00280	*welB*; alpha-L-Rha alpha-1,3-L-rhamnosyltransferase	*wclB;* glycosyltransferase (putative cell wall polysaccharide biosynthesis)	Sugar transferase	Conservation	Kankainen *et al.,* 2009 [44]
LGG_00281	*welC*; alpha-L-Rha alpha-1,3-L-rhamnosyltransferase	*wclC;* glycosyltransferase (putative cell wall polysaccharide biosynthesis)	Sugar transferase	Conservation	Kankainen *et al.,* 2009 [44]
LGG_00283*	*eps2*; hypothetical protein	*wclD;* putative glycosyltransferase (putative cell wall polysaccharide biosynthesis)	UDP-Glycosyltransferase	-	-
LGG_00295	Glycosyltransferase, group 2	Putative glycosyltransferase	Sugar transferase	Conservation	Kankainen *et al.,* 2009 [44]
LGG_00348	*yohJ*; lipopolysaccharide biosynthesis protein	Putative glycosyltransferase	UDP-Glycosyltransferase	Conservation	Kankainen *et al.,* 2009 [44]
LGG_00349	*yohH*; polyglycerol-phosphate alpha-glucosyltransferase	Putative glycosyltransferase	UDP-Glycosyltransferase	Conservation	Kankainen *et al.,* 2009 [44]
LGG_00645	Glycosyltransferase, group 2	Putative glycosyltransferase	Sugar transferase	Conservation	Kankainen *et al.,* 2009 [44]
LGG_00695	*gtrB*; glycosyltransferase, group 2	Putative glycosyltransferase	Sugar transferase	Conservation	Kankainen *et al.,* 2009 [44]
LGG_00794	*pbp1B*; penicillin-binding protein 1B	*pbpb1B;* putative glycosyltransferase, penicillin-binding protein 1B (peptidoglycan biosynthesis)	Pfam/CAZy	Conservation	Kankainen *et al.,* 2009 [44]
LGG_00825	*rfaG*; glycosyltransferase, group 1	Putative glycosyltransferase	UDP-Glycosyltransferase	Conservation	Kankainen *et al.,* 2009 [44]
LGG_00826	*cpoA*; glycosyltransferase, group 1	Putative glycosyltransferase	UDP-Glycosyltransferase	Conservation	Kankainen *et al.,* 2009 [44]
LGG_00928*	*yvcK*; transporter	Putative glycosyltransferase	Rossmann-fold domains	-	-
LGG_00985*	Integral membrane protein	Putative glycosyltransferase	Pfam/CAZy	-	-
LGG_00998	*arbX*; lipopolysaccharide biosynthesis glycosyltransferase	Putative glycosyltransferase	Sugar transferase	Conservation	Kankainen *et al.,* 2009 [44]
LGG_00999	*arbY*; lipopolysaccharide biosynthesis glycosyltransferase	Putative glycosyltransferase	Rossmann-fold domains	Conservation	Kankainen *et al.,* 2009 [44]
LGG_01057	Glycosyltransferase, group 2	Putative glycosyltransferase	Sugar transferase	Conservation	Kankainen *et al.,* 2009 [44]
LGG_01062^#^	*galU*; UTP-glucose-1-phosphate uridylyltransferase	UTP-glucose-1-phosphate uridylyltransferase	Sugar transferase	Conservation	Kankainen *et al.,* 2009 [44]
LGG_01069	*gtrB*; glycosyltransferase, group 2	Putative glycosyltransferase	Sugar transferase	Conservation	Kankainen *et al.,* 2009 [44]
LGG_01147	Glycosyltransferase, group 1	Putative glycosyltransferase	Rossmann-fold domains	Conservation	Kankainen *et al.,* 2009 [44]
LGG_01195*	*metQ*; ABC transporter	ABC transporter, putative bifunctional glycosyltransferase	Pfam	-	-
LGG_01283	*murG*; undecaprenyldiphospho-muramoylpentapeptide beta-N- acetylglucosaminyltransferase	*murG;* undecaprenyldiphospho-muramoylpentapeptide beta-N-acetylglucosaminyltransferase (peptidoglycan biosynthesis)	UDP-Glycosyltransferase	Conservation	Mengin-Lecreulx *et al.,* 1991 [26]; Kankainen *et al.,* 2009 [44]
LGG_01412*	*trmFO*; tRNA uracil-5-methyltransferase	tRNA uracil −5-methyltransferase, putative bifunctional glycosyltransferase	Rossmann-fold domains	-	-
LGG_01487	*pbp1A*; penicillin-binding protein 1A	*pbp1A;* putative glycosyltransferase, penicillin-binding protein 1A (peptidoglycan biosynthesis)	Pfam/CAZy	Conservation	Kankainen *et al.,* 2009 [44]
LGG_01538	Phage-related glycosyltransferase	Putative glycosyltransferase	Sugar transferase	Conservation	Kankainen *et al.,* 2009 [44]
LGG_01586	*yohH*; glycosyltransferase, group 1	Putative glycosyltransferase	UDP-Glycosyltransferase	Conservation	Kankainen *et al.,* 2009 [44]
LGG_01587	*yohJ*; glycosyltransferase, group 1	Putative glycosyltransferase	Rossmann-fold domains	Conservation	Kankainen *et al.,* 2009 [44]
LGG_01783	*pbp2A*; membrane carboxypeptidase, penicillin-binding protein 2A	*pbp2A;* bifunctional membrane carboxypeptidase, putative glycosyltransferase, penicillin-binding protein 2A (peptidoglycan biosynthesis)	Pfam/CAZy	Conservation	Di Guilmi *et al.,* 2003 [17]; Kankainen *et al.,* 2009 [44]
LGG_01991*	UDP-N-acetylglucosamine 2-epimerase	Epimerase, putative bifunctional glycosyltransferase	UDP-Glycosyltransferase	-	-
LGG_01992*	UDP-N-acetylglucosamine 2-epimerase	Epimerase, putative bifunctional glycosyltransferase	Sugar transferase	-	-
LGG_01999	*rmlA*; glucose-1-phosphate thymidylyltransferase	*rmlA*; glucose-1-phosphate thymidylyltransferase	Sugar transferase	Conservation	Kankainen *et al.,* 2009 [44]
LGG_02004	*eps3*; sugar or lipopolysaccharide synthesis transferase	Putative glycosyltransferase	Pfam	Conservation	Kankainen *et al.,* 2009 [44]
LGG_02023^#^	*glgP*; glycogen starch alpha-glucan phosphorylase	*glgP,* glycogen alpha-glucan phosphorylase	UDP-Glycosyltransferase	Conservation	Kankainen *et al.,* 2009 [44]
LGG_02024	*glgA*; glycogen synthase	*glgA;* glycogen synthase (glycogen biosynthesis)	UDP-Glycosyltransferase	Conservation	Kiel *et al.,* 1994 [42]; Kankainen *et al.,* 2009 [44]
LGG_02025^#^	*glgD*; glucose-1-phosphate adenylyltransferase	*glgD;* glucose-1-phosphate adenylyltransferase (glycogen biosynthesis)	Sugar transferase	Conservation	Ballicora *et al.*, 2003 [56]; Kankainen *et al.,* 2009 [44]
LGG_02026^#^	*glgC*; glucose-1-phosphate adenylyltransferase	*glgC;* glucose-1-phosphate adenylyltransferase (glycogen biosynthesis)	Sugar transferase	Conservation	Ballicora *et al.*, 2003 [56]; Kankainen *et al.,* 2009 [44]
LGG_02040^$^	*rmlA1*; glucose-1-phosphate thymidyl transferase	*rmlA1;* glucose-1-phosphate thymidyl transferase	Sugar transferase	Conservation	Kankainen *et al.,* 2009 [44]
LGG_02042	*rmlA2*; glucose-1-phosphate thymidylyltransferase	*rmlA2;* glucose-1-phosphate thymidylyltransferase	Sugar transferase	Conservation	Kankainen *et al.*, 2009 [44]
LGG_02043	*welE*; undecaprenyl-phosphate beta-glucosephosphotransferase	*welE;* priming glycosyltransferase (galactose-rich EPS biosynthesis)	Pfam	Experimental validation	Lebeer *et al.*, 2009 [37]
LGG_02044	*welF*; glycosyltransferase, group 1	*welF;* putative glycosyltransferase (galactose-rich EPS biosynthesis)	UDP-Glycosyltransferase	Conservation	Kankainen *et al.,* 2009 [44]
LGG_02045	*welG*; glycosyltransferase, galactofuranosyltransferase	*welG;* putative glycosyltransferase (galactose-rich EPS biosynthesis)	UDP-Glycosyltransferase	Conservation	Kankainen *et al.,* 2009 [44]
LGG_02046	*welH*; alpha-L-Rha alpha-1,3-L-rhamnosyltransferase	*welH;* putative glycoysltransferase (galactose-rich EPS biosynthesis)	Sugar transferase	Conservation	Kankainen *et al.,* 2009 [44]
LGG_02047	*welI*; glycosyltransferase, group 1	*welI*; glycosyltransferase (galactose-rich EPS biosynthesis)	UDP-Glycosyltransferase	Experimental validation	This work
LGG_02284	Glycosyltransferase, group 1	Putative glycosyltransferase	UDP-Glycosyltransferase	Conservation	Kankainen *et al.,* 2009 [44]
LGG_02285	*yohH*; glycosyltransferase, group 1	Putative glycosyltransferase	UDP-Glycosyltransferase	Conservation	Kankainen *et al.,* 2009 [44]
LGG_02347*	Hypothetical protein	Putative glycosyltransferase	Pfam	-	-
LGG_02562^#^	*glmU*; UDP-N-acetylglucosamine pyrophosphorylase	UDP-N-acetylglucosamine pyrophosphorylase	Sugar transferase	Conservation	Kankainen *et al.,* 2009 [44]
LGG_02869	Glycosyltransferase, group 1	Putative glycosyltransferase	UDP-Glycosyltransferase	Conservation	Kankainen *et al.,* 2009 [44]

**Locus tag**: gene identifier of the predicted GT. Genes for which a GT activity was predicted in this study that was not present in the current annotation are marked with a star (*). Potential false positive results are indicated with a hash (^#^). **Current annotation**: functional annotation as in current genome release of GenBank (NC_013198.1). **Proposed annotation**: new annotation proposed based on the results of our analysis. **HMM**: description of the Hidden Markov Model (HMM) with which the indicated GT was identified. **Evidence**: Level of evidence for the GT activity. Conservation: shows significant sequence conservation with an experimentally validated GT in a closely related species. Experimental validation: the GT activity has been experimentally validated in *Lactobacillus rhamnosus* GG. **Reference**: reference to the publication(s) supporting the evidence.

The performance of our GT prediction flow with and without the fold recognition filtering step was also evaluated in terms of the true-positive rate on the *C. jejuni* benchmark (containing 10 proteins with experimentally validated GT activity in *C. jejuni* NCTC 11168, see Methods). To obtain a full recall of 100% (that is retrieving all 10 positives), we had to make 184 predictions before the filtering. After the filtering the true positive rate increased from 10/184 to 10/44 (Additional file 1: Table S1). In addition to recovering all benchmark GTs (those indicated with experimental validation in Additional file 1: Table S1), most other predictions corresponded to previously made GT related annotations in *C. jejuni* NCTC 11168 that were based on indirect evidence (e.g. through experimental validation in other closely related species), such as the loci comprising the GT genes responsible for the synthesis of LOS (*CJ1133 – CJ1148*) [38], the GTs for *N-* (*CJ1121c– CJ1129c*) [33] and *O-*glycoprotein biosynthesis (*CJ1311* – *CJ1333*) [34] and the CPS biosynthesis cluster (*CJ1416c* – *CJ1442c*) [39, 40]. In addition, we made a total of 17 new predictions for yet unannotated genes in *C. jejuni* NCTC 11168 (Additional file 1: Table S1). Finally, we also retrieved four potential false positives (Additional file 1: Table S1).

The good agreement between our predictions and known information on glycosylation in *C. jejuni* NCTC 11168 [33], suggests that also for *L. rhamnosus* GG, the predictions summarized in Table 2 reflect true GTs. In addition, Table 2 provides a curated annotation update of GTs in *L. rhamnosus* GG: besides adding novel predictions, we removed potential erroneous annotations that originated through homology-based associations (indicated by conservation in Additional file 1: Table S1) as especially for GTs it is difficult to extrapolate the functional annotation without further experimental evidence (e.g. for *LGG_00279*). For GTs putatively involved in polysaccharide biosynthesis (*LGG_00279-LGG_00283*, see below), gene names were corrected in agreement with the conventional gene nomenclature [35].

Of the total number of 48 final predictions in *L. rhamnosus* GG (Table 2), five correspond to the experimentally documented locus encoding the enzymes involved in the synthesis of the complex galactose-rich EPS of *L. rhamnosus* GG [37, 41]. We also recovered the conserved cluster of GTs involved in the production of the intracellular storage glycogen-like polysaccharides [42] and the GTs necessary for the biosynthesis of PG [17]. In 33 cases, our predictions were consistent with previously annotated GTs (supported either by sequence conservation or by experimental evidence in related species. In five cases, indicated in Table 2 with a hash, our predictions are likely false positives. Eight of the 48 predicted GTs in *L. rhamnosus* GG were completely novel (indicated with a star in Table 2).

Among the novel predictions, two resulted from the screening with the ‘Rossmann-fold domain’ (class I) (*LGG_01412* and *LGG_00928*, see Table 2). The other novel predictions *LGG_01195* (previously annotated as ‘ABC transporter’), *LGG_00985* (previously annotated as ‘integral membrane protein’) and *LGG_02347* (previously annotated as ‘hypothetical protein’ were all detected by screening with the dedicated HMMs of class III (Table 1), further confirming the added value of these HMMs to find additional GTs. The screening with the HMMs of class II predicted as potential GTs LGG_00283 (a yet unannotated protein), LGG_01991 and LGG_01992. Both latter enzymes exhibit a high similarity with experimentally validated GTs in *E. coli* of the UDP-glycosyltransferase/Glycogen phosphorylase superfamily [42], further confirming their GT activity. However, they also show high sequence homology with UDP-*N*-acetylglucosamine 2-epimerases. This would be in agreement with the work of Campbell *et al.* (2000) showing that UDP-N-acetylglucosamine 2-epimerase has homology to phosphoglycosyl transferases and shares the same catalytic mechanism [43].

Despite the similar number of predicted GTs, the genomic organization of these predicted GTs is very different in *C. jejuni* NCTC 11168 and *L. rhamnosus* GG. In *C. jejuni* NCTC 11168, about 82% of the predicted GTs (corresponding to 36 GTs) are clustered into seven genomic regions, each of which contains at least two and on average five GTs that are physically located next to each other. The remaining eight predicted *C. jejuni* NCTC 11168 GTs are scattered in the genome (i.e. with no other GT present immediately up- or downstream)*.* For *L. rhamnosus* GG, a smaller fraction of the predicted GTs is organized in clusters: about 56% of the predicted GTs (corresponding to 28 GTs) are located in 9 clusters, that are on average slightly smaller (with a mean size of three GTs) than those found in *C. jejuni* NCTC 11168. The remaining 20 predicted GTs in *L. rhamnosus* GG are isolated in the genome. For both species, most of the well-studied experimentally verified GTs are localized in these clusters, e.g. in *C. jejuni* NCTC 11168 these clusters correspond to the genomic regions involved in the synthesis of LOS, CPS and *N-* and in *O-*protein glycosylation [4], whereas in *L. rhamnosus* GG one of the predicted clusters correspond to the known region for galactose-rich EPS [37, 41] and one to the cluster for the biosynthesis of intracellular storage glycogen-like polysaccharides [42, 44]. The function of the remaining seven clusters in *L. rhamnosus* GG is yet unknown.

Compared to the ones organized in clusters in both genomes, most of the GTs found in isolation appear to be much less studied. A closer inspection of these isolated GTs showed that in *L. rhamnosus* GG (in 7 of the 20 cases (*LGG_01057, LGG_01069, LGG_01147, LGG_01412, LGG_01487, LGG_01538, LGG_02004*)), but not in *C. jejuni* NCTC 11168, these isolated GTs are flanked by DNA topoisomerases, tyrosine recombinases, Holliday junction-specific endonucleases, phage-related resolvases and transposases (according to the current genome annotation of *L. rhamnosus* GG (NC_013198.1)). In addition, overlaying our predictions with the results of a previous comparative analysis between *L. rhamnosus* GG and its close relative *L. rhamnosus* LC705 [44], indicates that many of the isolated GTs we identified are specific for *L. rhamnosus* GG (such as *LGG_02004*). These observations, together with the lower fraction of GTs occurring in large genomic clusters, indicates that in *L. rhamnosus* GG, much more than in *C. jejuni* NCTC 11168, the glycosylation potential has been shaped by horizontal gene transfer and intra-genomic rearrangements, similarly to what has been observed for GTs belonging to family 6 of GTs in bacteria and vertebrates (CAZy database) [45, 46].

### Network-based strategy relating GTs to their substrate classes

To relate the predicted GTs to their potential substrates, we exploit the ‘local neighborhood’ of these GTs in a functional network, hereby assuming that GTs should be connected to their substrates, either directly or indirectly, via other GTs or enzymes. For the network, we relied on STRING, of which the functional interactions are inferred from physical (genome-wide protein-protein interactions, literature) and functional data (genomic co-localization, co-expression, co-occurrences, gene fusion-fission events) [14, 31]. The local neighborhood of a predicted GT (or local subnetwork) is here defined as the nodes that directly connect to the predicted GT (the latter of which is also referred to as the query GT) in the STRING network. We could derive 44 subnetworks for *C. jejuni* NCTC 11168, and 48 for *L. rhamnosus* GG. For each GT-specific subnetwork, the GO categories that were most overrepresented amongst the members of the subnetwork were used to infer for the query GT of each subnetwork a putative substrate class. As such we could predict a substrate class for 30/44 GTs in *C. jejuni* NCTC 11168 and for 20/48 GTs in *L. rhamnosus* GG which related to either saccharides, PG, proteins and lipids (see Additional file 2: Table S2 for *C. jejuni* NCTC 11168 and Table 3 for *L. rhamnosus* GG).

**Table 3:** **Proposed substrate classess of predicted glycosyltransferases in** ***Lactobacillus rhamnosus*** **GG**

Query-GT locus tag	Query-GT localization	Enriched GO categories	Membrane association	Partner GTs	Proposed substrate class of the query-GT	Potential protein substrate	Evidence	Reference
LGG_00280	C	EPS biosynthesis	LGG_00278 (hypothetical protein)	LGG_02043 LGG_00281 LGG_00283 LGG_00295 LGG_00279 LGG_01999	Extracellular saccharides	-	Conservation	Kankainen *et al.,* 2009 [44]
LGG_00281	C	EPS biosynthesis; PS transport	LGG_00278 (hypothetical protein)	LGG_00280 LGG_00295 LGG_01057 LGG_00279	Extracellular saccharides	-	Conservation	Kankainen *et al.,* 2009 [44]
LGG_00295	C	EPS biosynthesis	LGG_00296 (integral membrane protein)	LGG_00280 LGG_02043 LGG_00281 LGG_02869 LGG_01057	Extracellular saccharides	-	Conservation	Kankainen *et al.,* 2009 [44]
LGG_01062*	C	EPS biosynthesis	-	LGG_02026 LGG_02023 LGG_02025	Extracellularsaccharides	-	-	-
LGG_02040^$^	C	EPS biosynthesis; nucleotide-sugar metabolism	-	LGG_02042 LGG_02046	Extracellular saccharides	-	Conservation	Kankainen *et al.,* 2009 [44]
LGG_02042	C	EPS biosynthesis; nucleotide-sugar metabolism	-	LGG_02040	Extracellularsaccharides	-	Conservation	Kankainen *et al.,* 2009 [44]
LGG_02043	TM	Peptidyl-tyrosine dephosphorylation, regulation of catalytic acitivity, EPS biosynthesis	-	LGG_01992 LGG_02047	Extracellularsaccharides	-	Experimental validation	Lebeer *et al.,* 2009 [37]; Kankainen *et al.,* 2009 [44]
LGG_02045	C	Polysaccharide biosynthesis; polysaccharide transport	LGG_00282 (polysaccharide transporter)	LGG_00998 LGG_00999 LGG_02046 LGG_02047	Extracellular saccharides	-	Conservation	Kankainen *et al.,* 2009 [44]
LGG_02046	C	EPS biosynthesis; polysaccharide transport	LGG_02049 (polysaccharide transporter)	LGG_02045 LGG_02047 LGG_01999	Extracellular saccharides	-	Conservation	Kankainen *et al.,* 2009 [44]
LGG_02047	C	Polysaccharide biosynthesis; polysaccharide transport	LGG_02043 (undecaprenyl-P-β-glucosephosphotransferase)	LGG_02043 LGG_02045 LGG_02046 LGG_02869 LGG_00295 LGG_01057	Extracellular saccharides	-	Experimental validation	This work
LGG_01062*	C	Glycogen biosynthesis	-	LGG_02026 LGG_02023 LGG_02025	Intracellular saccharides	-	-	-
LGG_02023	C	Glycogen biosynthesis; pyrimidine nucleoside metabolism	-	LGG_02026 LGG_01062 LGG_02024 LGG_02025	Intracellular saccharides	-	Conservation	Kankainen *et al.,* 2009 [44]
LGG_02024	C	Glycogen biosynthesis; response to antibiotic	-	LGG_02023 LGG_02025 LGG_02026	Intracellular saccharides	-	Conservation	Kiel *et al.,* 1994 [42]; Kankainen *et al.,* 2009 [44]
LGG_02025	C	Glycogen biosynthesis	-	LGG_02023 LGG_02024 LGG_02026	Intracellular saccharides	-	Conservation	Ballicora *et al.,* 2003 [56]; Kankainen *et al.,* 2009 [44]
LGG_02026	C	Glycogen biosynthesis	-	LGG_02023 LGG_02024 LGG_02025	Intracellular saccharides	-	Conservation	Ballicora *et al.,* 2003 [56]; Kankainen *et al.,* 2009 [44]
LGG_00998	C	Carbohydrate metabolism; lipids metabolism	LGG_00995 (hypothetical protein)	LGG_02045 LGG_00999	Lipid	-	Conservation	Kankainen *et al.,* 2009 [44]
LGG_00999	C	Carbohydrate metabolism; lipids metabolism	LGG_00995 (hypothetical protein)	LGG_02045 LGG_00998	Lipid	-	Conservation	Kankainen *et al.,* 2009 [44]
LGG_01057*	C	Carbohydrate metabolism; lipids metabolism	LGG_02004 (sugar or LPS synthesis transferase)	LGG_02004 LGG_00280 LGG_02043 LGG_02869 LGG_00295 LGG_02046 LGG_02047	Lipid	-	-	-
LGG_00794	TM	PG-based cell wall biogenesis	-	-	Peptidoglycan	-	Conservation	Kankainen *et al.,* 2009 [44]
LGG_01283	C	PG-based cell wall biogenesis	LGG_01192 (rod shape-determining protein RodA)	LGG_01487	Peptidoglycan	-	Conservation	Mengin-Lecreulx *et al.,* 1991 [26]; Kankainen *et al.,* 2009 [44]
LGG_01487	TM	PG-based cell wall biogenesis	-	LGG_01283	Peptidoglycan	-	Conservation	Kankainen *et al.,* 2009 [44]
LGG_01538*	TM	PG biosynthetic process; regulation of cell shape; dephosphorylation; response to antibiotics	-	LGG_00280	Peptidoglycan	-	-	-
LGG_01783	TM	PG-based cell wall biogenesis	*-*	-	Peptidoglycan	*-*	Conservation	Di Guilmi *et al.,* 2003 [17]; Kankainen *et al.,* 2009 [44]
LGG_00794*	TM	Regulation of cell shape; cell cycle	*-*	*-*	Protein	LGG_01280 (cell division protein FtsI)	-	-
LGG_00825*	C	Protein translation	LGG_00751 (SNARE associated golgi protein)	LGG_00826	Protein	LGG_00829 (YkuJ protein)	-	-
LGG_00826*	C	Protein translation; amino acid transport	LGG_00751 (SNARE associated golgi protein)	LGG_00825 LGG_02047	Protein	LGG_00829 (YkuJ protein)	-	-
LGG_01147*	C	DNA metabolic process	LGG_01146 (predicted ORF)	-	Protein	LGG_01145 (DNA-entry nuclease)	-	-
LGG_01283*	C	Regulation of cell shape; response to antibiotic, cell division	LGG_01192 (rod shape-determining protein RodA)	LGG_01487	Protein	LGG_01280 (cell division protein FtsI)	-	-
LGG_01487*	TM	Regulation of cell shape; cell division	-	LGG_01283	Protein	LGG_01706 (cell division protein/penicillin-binding protein 2); LGG_01280 (cell division protein FtsI); LGG_00254 (D-alanyl-D-alanine carboxypeptidase)	-	-
LGG_01783*	TM	Regulation of cell shape; cell cycle	-	-	Protein	LGG_01280 (cell division protein FtsI)		

**Locus tag**: gene identifier of the predicted GT used as query in STRING to obtain a query-dependent subnetwork. **Localization**: indicates whether the query-GT was predicted to be a cytoplasmic (C) or a transmembrane protein (TM). **Enriched GO categories**: GO categories enriched amongst the members of the query-dependent subnetwork of the indicated query-GT. Only categories showing an enrichment value of p < 0.05 are shown (according to a hypergeometric test corrected for multiple testing using False Discovery Rate). **Membrane association**: refers to edges between the query-GT and members of its subnetwork predicted to be transmembrane proteins. **Partner GTs**: predicted/experimentally validated GTs that belong to the subnetwork of the query-GT. **Proposed substrate class of the query-GT**: inferred from the GO enrichment analysis of the query-dependent subnetwork of the indicated query-GT derived from STRING. Novel substrate predictions derived from this study are indicated by a star (*) next to the locus tag of the corresponding query-GT. **Potential protein substrate**: it refers to edges between the query-GT and members of its subnetwork predicted to have *N*- or *O*-glycosylation sites. Such proteins are therefore suggested to be potential substrates of the query-GT in the cases where proteins are the proposed substrate. **Evidence**: level of evidence for the predicted substrate class of the query-GT. Conservation: shows significant sequence conservation with a GT for which the substrate specificity has been experimentally validated in closely related species. Experimental validation: the substrate specificity of the GT has been experimentally validated in *Lactobacillus rhamnosus* GG. **Reference**: publication(s) supporting the predicted substrate class of the query-GT.

The relation of the predicted GTs with their network neighbours was further specified using information on putative membrane associations or presence of glycosylation sites in the network members (Methods): a query-GT being connected to a transmembrane protein is referred to as a ‘membrane association’ and is indicative for soluble GTs that exert their action by interacting with transmembrane proteins, e.g. a transporter of glycoconjugates [47–51]. A query-GT being connected to proteins with putative glycosylation sites hints towards the glycosylation of those proteins by the query-GT (substrate relation).

**Figure 3:**
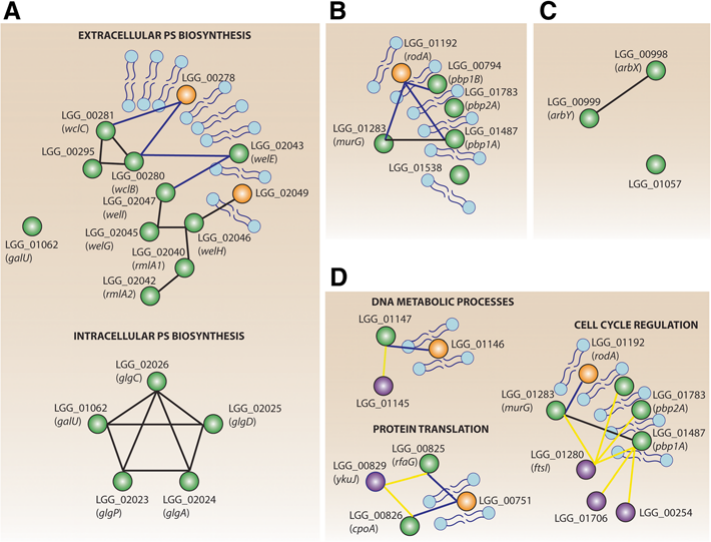
**Consensus networks derived for each of the predicted substrate classes of putative GTs in** ***L. rhamnosus*** **GG.** Consensus networks show all GTs, having the same substrate class, together with their protein neighbors that are hypothesized to contribute to the same common glycosylation mechanism as the one the GTs are involved in. On the consensus networks, nodes are proteins than can either be GTs (green nodes), transmembrane proteins (orange nodes) or proteins containing glycosylation signals (violet nodes). Membrane associations established between GTs and transmembrane proteins are represented by blue edges while predicted substrate relations between GT and proteins containing glycosylation signals are represented by yellow edges. Black edges refer to interactions between predicted GTs. If the local network neighborhood of GTs (local subnetwork) belonging to the same substrate class shows enrichment in more than one GO category (e.g. both the GO terms of EPS and glycogen biosynthesis), the consensus network is shown for each of the enriched GO categories. **A**: consensus networks involving GTs, predicted to glycosylate saccharides. Note that here two independent consensus networks were derived corresponding to respectively extracellular and intracellular PS biosynthesis. **B**: consensus network involving GTs, predicted to glycosylate peptidoglycan (PG). **C**: consensus network involving GTs, predicted to glycosylate lipids. **D**: consensus networks involving GTs, predicted to glycosylate proteins. Three independent consensus networks were derived corresponding to respectively cell cycle regulation, protein translation and DNA metabolic processes. Our analysis suggests substrate promiscuity for MurG, PBP1A, PBP1B and PBPA, all of which were predicted to be involved in the glycosylation of both peptidoglycan and proteins.

To gain insight in the mutual interactions between GTs and of these GTs with other proteins involved in the same process, we created ‘consensus networks’ that combine the local network neighbourhood of all GTs, predicted to belong to the same specificity class and of which the local subnetworks are enriched in the same GO terms (Figure 3).

### Inferred substrate classes of predicted GTs in the benchmark

To assess the extent to which our network-based approach was able to correctly infer substrate classes, we used as benchmark again the 10 GTs in *C. jejuni* NCTC 11168 for which also the substrate specificity is known (see Methods). Our strategy was able to recover the known substrate class of all 10 GTs (sensitivity of 100%) on a total of 31 predicted substrate classes for GTs in *C. jejuni* (true positive rate of 10/31).

### Inferred substrate classes of predicted GTs in *L. rhamnosus* GG

The 20 GTs in *L. rhamnosus* GG for which we could predict their putative substrate class are summarized in Table 3.

#### GTs predicted to glycosylate saccharides

In *L. rhamnosus* GG, the substrate class saccharides (Figure 3A) comprises the largest number of GTs, which is to be expected as saccharides are the most common substrates for GTs [3]. The group of GTs that could be related to saccharides comprises two consensus networks: the first consensus network consists of GTs that, according to their GO annotation are involved in the biosynthesis of extracellular polysaccharides (WclC, WclB, WelE, WelG, WelH, WelI, RmlA2, LGG_00295) [37, 41]. The topology of this consensus network is indicative for *en bloc* glycosylation [4, 5] because it contains several interconnected soluble GTs, all linked to a membrane-bound priming GT together with Wzx flippases that transfer the subunits *en bloc* (see below).

This consensus network (Figure 3A) can be further subdivided into two cliques of interconnected GTs. The first clique (*welI-welG-welH-rmlA*1-*rmlA*2) contains genes involved in the synthesis of galactose-rich EPS, such as amongst others WelE (LGG_02043), the priming GT, with an experimentally verified substrate [37]. From the previously annotated gene cluster for galactose-rich EPS [37, 44], our analysis only missed *welJ*, annotated as alpha-1,3-galactosyltransferase (*LGG_02048*), as this gene was not predicted as a GT in our analysis. This gene does not appear to contain any signatures of the currently known HMMs for GTs and might represent a false negative of our analysis or an erroneous annotation in the current release of the *L. rhamnosus* GG genome NC_013198.1. This last hypothesis is supported by the small gene size of *welJ*, which would be atypical for a GT.

Regarding the second clique (*wclC-LGG_00295-wclB*), it contains genes for which the substrate specificity towards saccharides is known from homology-based extrapolation only. As we know from previous work that *L. rhamnosus* GG contains, besides its galactose-rich EPS also shorter, glucose-rich polysaccharides structures, we would hypothesize that this clique contains the missing genes for those glucose-rich polysaccharides structures [52]. The prediction of an independent Wzx flippase for each of the sets of interconnected GTs (cliques) (i.e. LGG_02049 for the galactose-rich clique and WclC and WclB for the clique putatively responsible for glucose-rich EPS synthesis), together with the known exquisite substrate specificity of Wzx flippases [53] further supports the hypothesis of each clique being responsible for the biosynthesis of another glycan type. Assuming that indeed the upper clique is involved in the synthesis of glucose-rich saccharide structures implies that the predicted link between WelE and this second clique (WclC, LGG_00295 and WclB) must be mere functional (i.e. not invoking a direct interaction), since knock-out experiments indicate that WelE is not the direct priming GT of the glucose-rich EPS structures [37].

The second consensus network (Figure 3A lower part, GlgA, GlgC, GlgD, GlgP, GalU) recapitulates all known members of the glycosylation system involved in glycogen synthesis except GlgB (LGG_02027), a conserved glycogen branching enzyme with transglycosylase activity, i.e. an enzyme that has both hydrolase and GT characteristics [54], which was not picked up by our HMM-based search step. From the predicted GTs in this network only GlgA, previously already known as a glycogen synthase, seems to be a genuine GT [42, 55]. For the other proteins GlgC, GlgD and GlgP, GalU -though related to glycan biosynthesis- enzyme activities other than GT activity have been documented [56]. The consensus network of the glycogen enzymes is composed solely of soluble proteins, which is in agreement with the intracellular nature of the glycogen-like polysaccharides. The connectivity between only soluble GTs points towards a sequential glycosylation mechanism in which sugar monomers are directly transferred from activated sugar-nucleotide donors (probably produced by GalU) to the respective substrates.

#### GTs predicted to glycosylate peptidoglycans

Five GTs could be related to PG precursors (PBP1A, PBP1B, PBP2A, MurG and LGG_01538), an annotation that has previously been suggested based on sequence conservation of these GTs across species (Figure 3B). GO enrichment analysis of their functional subnetworks suggests, both in *L. rhamnosus* GG (Table 3) and *C. jejuni* NCTC 11168 (Additional file 2: Table S2), a link between PG biosynthesis and a diverse set of processes, such as the regulation of cell shape, cell cycle and response to antibiotics, in agreement with the well-known functions of PG. Compared to the genes involved in EPS biosynthesis, it is remarkable that the GT genes involved in PG biosynthesis and remodelling do not occur in genomic clusters. The diversity of the processes in which these PG GTs are involved, might imply their necessity to be expressed under different environmental stimuli, which in turn can explain their organization in individual transcriptional units rather than in operons.

The consensus network of this class of GTs (Figure 3B) shows that all of these GTs are predicted to have transmembrane domains except for the soluble protein encoded by *murG*. The network organization is consistent with the known two-stage mechanism of bacterial PG biosynthesis consisting of cytoplasmic glycosylation reactions mediated by soluble GTs, followed by membrane-bound transglycosylation activities [57, 58].

#### GTs predicted to glycosylate lipids

The group of GTs that could be related to lipids contains three predicted GTs (LGG_00998, LGG_00999, LGG_01057) (Figure 3C). For these three GTs, their respective functional subnetworks showed enrichment for the terms ‘carbohydrate’ and ‘lipid metabolism’, suggesting that they are involved in the synthesis of lipoglycans present on the cell wall of the Gram-positive bacterium *L. rhamnosus* GG. This predicted role is more plausible than their homology based annotated role as ‘LPS biosynthesis glycosyltransferases’, as LPS molecules are absent in Gram-positives. The sparsity of the consensus network of these three GTs might be due to the incompleteness of the STRING network. So far, the existence of lipoglycans in *L. rhamnosus* GG has not yet been shown by biochemical studies.

#### GTs predicted to glycosylate proteins

A final group of seven GTs could be related to protein substrates and contains both predicted transmembrane (PBP1A, PBP1B, PBP2A) and predicted soluble GTs (LGG_00825, LGG_00826, LGG_01147, MurG). The GTs in this class were classified as protein GTs because the putative protein substrates in their subnetworks carry glycosylation signals. The GTs fall apart in three consensus subnetworks related to respectively cell cycle regulation, protein translation and DNA metabolic processes (Figure 3D).

**Figure 4:**
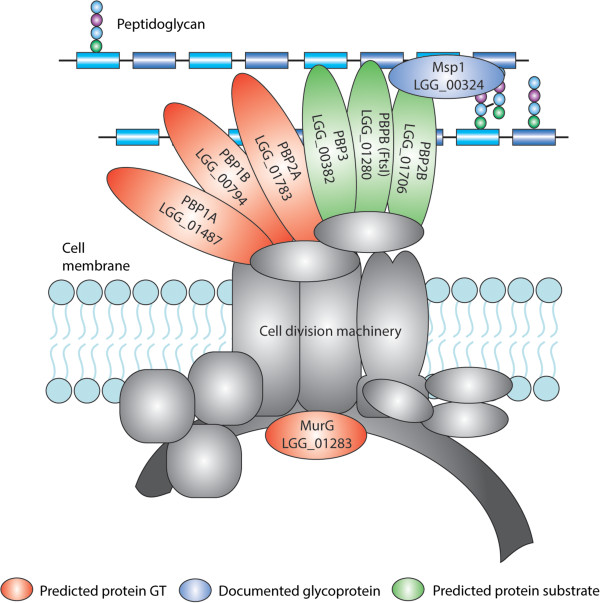
**Protein glycosylation of the cell division machinery.** Schematic overview of the cell division machinery of *L. rhamnosus*. PBP1A, PBP1B, PBPB2A and MurG are predicted to be putative GTs. Our network-based analysis predicted PBP3, FtsI and PBP2B as putative substrates of the indicated GTs. The Msp1 cell wall hydrolase is the experimentally validated glycoprotein in *L. rhamnosus* GG [36].

A first consensus network comprises three transmembrane GTs (PBP1A, PBP2A, PBP1B) and MurG all predicted to be involved in ‘cell cycle regulation’ (according to the GO enrichment analysis of their respective subnetworks). Their consensus network points towards a substrate relation between each of the four GTs MurG, PBP1A, PBP2A and PBP1B, and cell division proteins (between MurG, PBP1A, PBP2A and PBP1B and the cell division protein FtsI on the one hand and between PBP1A, LGG_01706 and LGG_00254 on the other hand). Two previous studies further support our predictions: in *Bacteroides fragilis* FtsI, and other cell cycle related proteins such as FtsX and FtsQ, have been shown to be glycosylated [59]*.* In addition, a very recent study in *L. plantarum* WCFS1 [60] provides experimental evidence for the glycosylation of the cell division proteins FtsY, FtsZ, and FtsK 1 [60]. Our results – on the other hand- indicate that the three transmembrane GTs and MurG, known to be involved in PG biosynthesis show substrate promiscuity and would also have relations with protein substrates in *L. rhamnosus* GG (Table 3). A link between PG biosynthesis and protein glycosylation is not completely impossible given the fact that these predicted ‘promiscuous’ GTs co-occur with their predicted protein substrates including FtsI in cell division multi-enzyme complexes (Figure 4).

This link between PG biosynthesis and protein glycosylation is further supported by the fact that the other predicted protein substrate of PBP1A (the D-alanyl-D-alanine carboxypeptidase (LGG_00254)), is also known to be directly involved in PG biosynthesis by introducing interpeptide cross-links. Although not yet reported for D,D trans-peptidases, other PG remodeling enzymes such as the PG hydrolases Msp1 in *L. rhamnosus* GG [36] and Acm2 in *L. plantarum* WCFS1 [61] were recently shown to be glycosylated [62].

A second consensus cluster is composed of two soluble GTs predicted to be involved in ‘protein translation’ (LGG_00825-LGG_00826). Both of these GTs were predicted to participate in the glycosylation of YkuJ, a protein co-translated with CcpC, a repressor of the tricarboxylic acid cycle in *Bacillus subtillis* (Figure 3C) [63]. LGG_00825 and LGG_00826 also exhibit a membrane association mediated by LGG_00751, annotated in *L. rhamnosus* GG as a hypothetical protein with a pfam09335 domain typical for SNARE associated Golgi proteins in eukaryotes. The membrane association of both GTs via a protein involved in translation, together with the fact that the subnetwork of LGG_00825 is enriched in the function ‘protein translation’ is consistent with the existence of an eukaryotic counterpart of sequential co-translational glycosylation in bacteria [51].

A last consensus network comprises only one GT, LGG_01147, predicted to be involved in ‘DNA metabolic processes’. LGG_01147 shows a substrate relation with LGG_01145, encoding a putative DNA entry nuclease, while establishing a membrane association mediated by LGG_01146 (Figure 3C). Little is known about these interacting partners, but nucleases are often glycosylated in eukaryotes [64]. Although not specifically related to nucleases, glycosylation of extracellular enzymes has been reported in prokaryotes [36, 61, 65–67] and is thought to promote their stability [36]. Whether this is also the case in LGG_01146 needs to be further substantiated.

### Experimental analysis of the GT network for EPS biosynthesis

**Figure 5:**
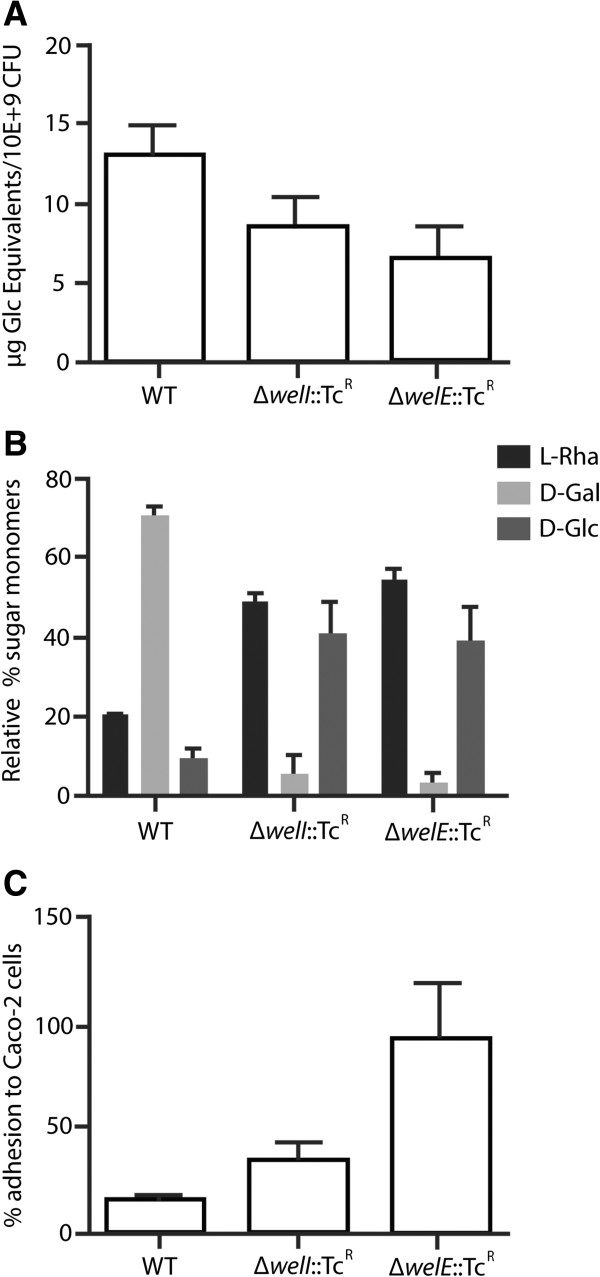
**Experimental validation of the EPS network hierarchy. A**: Total cell wall polysaccharides were extracted from respectively LGG wild-type, a Δ*welE*::Tc^R^ gene deletion mutant (CMPG5351) and Δ*welI*::Tc^R^ gene deletion mutant (CMPG10811). The total amount of EPS was measured. Error bars indicate standard deviations (of three repeats). One-way ANOVA statistical analysis rendered a p-value smaller than 0.05 for the variation of EPS across strains. **B**: Sugar monomer composition. The data are expressed as relative amounts, taking the total amount of detected monomeric sugars as 100%. Error bars indicate standard deviations (of three repeats). One-way ANOVA analyses (performed independently on each of the three datasets) rendered significant p-values (<0.05) for the variation of each sugar monomer across strains. **C**: Adhesion capacity. The adhesion capacity of wild type and mutants to Caco-2 cells is compared. Error bars indicate standard deviations (of three repeats). A One-way ANOVA analysis rendered a significant p-value (<0.05) for the variation of the adhesion capacity of the strains.

We experimentally validated the GT network hierarchy within the clique for galactose-rich EPS (Figure 3A) by constructing a gene deletion mutant in the *welI* gene and comparing its phenotype to the phenotypes of the wild type (WT) and the gene deletion mutant of the priming GT WelE. As phenotypes, we tested the amount and monomer composition of EPS, and the adhesion capacity to the intestinal epithelial cell line Caco-2 as an indirect measurement of the EPS level [37]. According to our predictions, WelI would be one of the GTs that transfer sugar moieties to the sugar subunit initiated by the priming GT WelE. Based on these predictions, a gene deletion mutant of WelI would be expected to affect the amount of EPS, as in the absence of WelI less sugar moieties will be transferred to the subunit initiated by the WelE, but the effect of the WelI deletion on the phenotype should be less severe than the effect observed when deleting the priming GT WelE. A phenotype for the *welI* mutant intermediate between the WT and the *welE* gene deletion mutant is indeed observed for both assays confirming the predicted role of WelI upstream of WelE: the Δ*welI*::Tc^R^ mutant displays a lower galactose-rich EPS content than the WT, but a higher content and more galactose than the gene deletion mutant of the priming GT WelE (Figure 5A and B). In agreement with EPS having a negative effect on adhesion, the adherence capacity is the highest for the *welE* mutant, intermediate for the *welI* mutant and lowest for the WT (Figure 5C).

## Discussion

In this work we developed an analysis flow that uses sequence-based strategies to predict novel GTs, but also exploits a network-based approach to infer the substrate classes of these putative GT. Using a broad definition of GT activity, including also HMMs for OSTs and other non-typical GTs, allowed covering a large part of the glycosylation potential. Applying our flow resulted in a careful revision of GTs in the current genome annotation of *L. rhamnosus* GG (NC_013198.1). We confirmed the identity of 33 GTs and predicted 8 novel ones. In contrast to what is observed in *C. jejuni* NCTC 11168, GTs appear to be much less clustered in genomic regions, but rather occur as isolated genes flanked by transposable elements. This points towards a key role of horizontal gene transfer in the acquisition of the glycosylation potential of *L. rhamnosus* GG.

Complementing the sequence-based with a network based-approach allowed us to also relate some of those GTs to their potential substrates. Most prior experimental studies focused on analyzing the specificity of GTs organized in clusters together with their auxiliary enzymes, as this allows for the straightforward extrapolation of known specificities of some members to all members in the cluster. By considering, next to the genomic organization, also links in a functional network, we could predict the substrate classes for the numerous, isolated GTs in *L. rhamnosus* GG. Exploiting membrane associations and substrate relations for the nodes in the GT-centered networks helped predicting the mutual relations between the GTs and between the GTs and their substrates.

Our analysis contributed to the annotation of GTs in *L. rhamnosus* GG. For instance, we hypothesize that one of the genomic regions that was previously annotated to be involved in EPS biosynthesis in general would contain the missing genes involved in the biosynthesis of short glucose-rich polysaccharides that are known to decorate the surface of *L. rhamnosus* GG [68]. In addition, we uncovered several novel interactions. For instance, for the isolated GTs known to be involved in PG biosynthesis (PBP1B, PBP2A, PBP1A and MurG), our network-based approach suggests an additional role in the glycosylation of proteins that are either involved in the biosynthesis of the PG (LGG_00254) or in cell division (LGG_01280 or FtsI). Substrate promiscuity of GTs is not uncommon in bacteria as for instance in Gram-negative pathogens, enzymes with relaxed specificity are shared between different processes, such as LPS and glycoprotein biosynthesis [4, 38]. Validating the activity of GTs that were predicted to glycosylate proteins- is cumbersome, as *in vitro* enzymatic assays do not represent the cellular conditions that are relevant for the assembly of these GTs in multi-enzyme membrane-associated complexes [69]. However, because PG biosynthesis is a process involving multi-enzyme complexes for which the assembly is tightly regulated [69], it is not unlikely that also protein glycosylation would act as an additional regulatory layer in this structural complex formation. Provided our hypothesis on their substrate specificity towards both proteins and PG would be true, these promiscuous GTs (PBP1B, PBP2A, PBP1A and MurG) are unlikely to be the priming GTs of their putative protein substrates, given their well characterized specificities towards PG precursors in both Gram-positives and negatives [70]. We hypothesize that the priming GTs predicted to be involved in protein glycosylation must be (*Lactobacillus*) species*-* or strain-specific rather than generally conserved in prokaryotes. This is supported by the observation that the best documented glycoprotein in *L. rhamnosus* GG, *i.e.* Msp1, another protein associated to the divisome [36] (see Figure 4), was no longer glycosylated after transfer to the Gram-negative *E. coli*[15] despite the fact that *E. coli* also has PBP1A, PBP1B, PBP2A and MurG homologs. In addition, the sugar monomers added on Msp1 [36] and related PG hydrolases such as Acm2 [71] show different sugar lectin specificities in *L. rhamnosus, L. casei* and *L. plantarum*.

## Conclusions

Our results show how combining sequence- and network-based computational predictions can unveil insights in the bacterial glycosylation potential, thereby providing novel links and interesting hypotheses for further investigation.

## Authors’ information

The authors wish it to be known that, in their opinion, Aminael Sánchez-Rodríguez and Hanne L.P. Tytgat should be regarded as joint first authors.

## Electronic supplementary material

Additional file 1: Table S1: List of glycosyltransferases predicted in the genome of *Campylobacter jejuni* NCTC 11168. **Locus tag**: gene identifier of the predicted GT. Genes for which a GT activity was predicted in this study that was not present in the current annotation are marked with a star (*). Potential false positive results are indicated with a hash (#). **Current annotation**: functional annotation as in the current genome release of GenBank (NC_002163.1). **Proposed annotation**: new annotation based on the results of our analysis. **HMM**: Description of the Hidden Markov Model (HMM) with which the indicated GT was identified. Note that all predicted GTs also passed the fold based filtering. **Evidence**: Type of evidence for the GT activity. Conservation: shows significant sequence conservation with an experimentally validated GT in a closely related species. Experimental validation: the GT activity has been experimentally validated in *Campylobacter jejuni* NCTC 11168. **Reference**: reference to the publication(s) supporting the prediction. (PDF 51 KB)

Additional file 2: Table S2: Proposed substrate classes of glycosyltransferases in *Campylobacter jejuni* NCTC 11168. **Locus tag**: gene identifier of the predicted GT used as query in STRING to obtain a query-dependent subnetwork. **Localization**: indicates whether the query-GT was predicted to be cytoplasmic (C) or a transmembrane protein (TM). **Enriched GO categories**: GO categories enriched amongst the members of the query-dependent subnetwork of the indicated query-GT. Only categories showing an enrichment value p < 0.05 are shown (according to a hypergeometric test corrected for multiple testing using False Discovery Rate). **Membrane association**: it refers to edges between the query-GT and members of its subnetwork predicted to be transmembrane proteins. **Partner GTs**: predicted/experimentally validated GTs that belong to the subnetwork of the query-GT. **Predicted substrate class of a query-GT**: inferred from the GO enrichment analysis of the query-dependent subnetwork of the indicated query-GT derived from STRING. **Potential protein substrate**: it refers to edges between the query-GT and members of its subnetwork predicted to have *N*- or *O*-glycosylation signals. Such proteins are therefore suggested to be potential substrates of the query-GT in the cases where proteins are the proposed substrate. **Evidence**: level of evidence for the substrate class prediction. **Conservation**: shows a significant sequence conservation with a GT for which a susbtrate specificity has been experimentally validated in a closely related species. Experimental validation: the substrate specificity of the GT has been experimentally validated in *Campylobacter jejuni* NCTC 11168. **Reference**: publication(s) supporting the predicted substrate class of the query-GT. (PDF 131 KB)
