# Supplementary material for: A network-based approach to identify substrate classes of bacterial glycosyltransferases
Source: BMC Genomics. 2014 May 8;15(1):349. doi: 10.1186/1471-2164-15-349 (PMC4039749; doi:10.1186/1471-2164-15-349)
Supplement: Supplementary file 1 — Additional file 1: Table S1: List of glycosyltransferases predicted in the genome of Campylobacter jejuni NCTC 11168. Locus tag: gene identifier of the predicted GT. Genes for which a GT activity was predicted in this study that was not present in the current annotation are marked with a star (*). Potential false positive results are indicated with a hash (#). Current annotation: functional annotation as in the current genome release of GenBank (NC_002163.1). Proposed annotation: new annotation based on the results of our analysis. HMM: Description of the Hidden Markov Model (HMM) with which the indicated GT was identified. Note that all predicted GTs also passed the fold based filtering. Evidence: Type of evidence for the GT activity. Conservation: shows significant sequence conservation with an experimentally validated GT in a closely related species. Experimental validation: the GT activity has been experimentally validated in Campylobacter jejuni NCTC 11168. Reference: reference to the publication(s) supporting the prediction. (PDF 51 KB) [file 12864_2013_6075_MOESM1_ESM.pdf]

Table S1. List of glycosyltransferases predicted in the genome of *Campylobacter jejuni* NCTC 11168. Locus tag: gene identifier of the predicted GT. Genes for which a GT activity was predicted in this study that was not present in the current annotation are marked with a star (*). Potential false positive results are indicated with a hash (#). **Current annotation**: functional annotation as in the current genome release of GenBank (NC_002163.1). **Proposed annotation:** new annotation based on the results of our analysis. **HMM**: Description of the Hidden Markov Model (HMM) with which the indicated GT was identified. Note that all predicted GTs also passed the fold based filtering. **Evidence:** Type of evidence for the GT activity. Conservation: shows significant sequence conservation with an experimentally validated GT in a closely related species. Experimental validation: the GT activity has been experimentally validated in *Campylobacter jejuni* NCTC 11168. **Reference**: reference to the publication(s) supporting the prediction.

Locus tag	Current Annotation	Proposed Annotation	HMM	Evidence	Reference
CJ0288c	*lpxB;* lipid-A-disaccharide synthase	*lpxB;* lipid-A-disaccharide synthase	UDP-Glycosyltransferase	Conservation	Gundogdu *et al*., 2007
CJ0497*	Lipoprotein	Putative glycosyltransferase	Sugar transferase	-	-
CJ0508	*pbpA;* penicillin-binding protein	*pbpA;* putative glycoysltransferase, penicillin-binding protein A (peptidoglycan biosynthesis)	Pfam/CAZy	Conservation	Murray *et al.,* 1997
CJ0707	*kdtA;* 3-deoxy-D-manno-octulosonic-acid transferase	*kdtA;* 3-deoxy-D-manno-octulosonic-acid transferase	UDP-Glycosyltransferase	Conservation	Clementz *et al.,* 1991
CJ0813#	*kdsB;* 3-deoxy-manno-octulosonate cytidylyltransferase	*kdsB;* 3-deoxy-manno-octulosonate cytidylyltransferase	Rossmann-fold domains	Conservation	Parkhill *et al.,* 2000
CJ0821*	*glmU;* bifunctional N-acetylglucosamine-1-phosphate uridyltransferase/glucosamine-1-phosphate acetyltransferase	Bifunctional N-acetylglucosamine-1-phosphate uridyltransferase/glucosamine-1-phosphate acetyltransferase, putative glycosyltransferase	Rossmann-fold domains	-	-
CJ1039	*murG;* UDP-diphospho-muramoylpentapeptide beta-N-acetylglucosaminyltransferase	*murG;* UDP-diphospho-muramoylpentapeptide beta-N-acetylglucosaminyltransferase (peptidoglycan biosynthesis)	Rossmann-fold domains	Conservation	Mengin-lecreulx *et al.,* 1991
CJ1121c#	*pglE;* UDP-4-keto-6-deoxy-GlcNac C4 aminotransferase	*pglE;* UDP-4-keto-6-deoxy-GlcNac C4 aminotransferase	Pfam/CAZy	Experimental validation (of other than the GT activity)	Vijayakumar *et al.,* 2006; Schoenhofen *et al.,* 2006
CJ1124c	*pglC;* galactosyltransferase	*pglC;* galactosyltransferase (Protein N-glycosylation)	Pfam/CAZy	Experimental validation	Glover *et al.,* 2006
CJ1125c	*pglA;* GalNAc transferase	*pglA;* GalNAc transferase (Protein N-glycosylation)	UDP-Glycosyltransferase	Experimental validation	Glover *et al.,* PNAS, 2005
CJ1126c	*pglB;* oligosaccharide transferase to N-glycosylate proteins	Oligosaccharide transferase to N-glycosylate proteins (Protein N-glycosylation)	Pfam/CAZy	Experimental validation	Glover *et al.,* Chem. Biol., 2005

CJ1127c	*pglJ;* GalNAc transferase	*pglJ;* GalNAc transferase (Protein N-glycosylation)	Sugar transferase	Experimental validation	Glover *et al.,* 2005b
CJ1128c	*pglI;* glycosyltransferase	*pglI;* glycosyltransferase (Protein N-glycosylation)	Sugar transferase	Experimental validation	Glover *et al.,* 2005b
CJ1129c	*pglH;* GalNAc transferase/polymerase	*pglH;* GalNAc transferase/polymerase (Protein N-glycosylation)	UDP-Glycosyltransferase	Experimental validation	Glover *et al.,* 2005b
CJ1133	*waaC;* heptosyltransferase I	*waaC;* heptosyltransferase I (LOS & CPS biosynthesis)	UDP-Glycosyltransferase	Experimental validation	Kanipes *et al.,* 2006
CJ1135	Glycosyltransferase	Putative glycosyltransferase (LOS biosynthesis)	Sugar transferase	Conservation	Taboada *et al,* 2007
CJ1136	Glycosyltransferase	Glycosyltransferase (LOS biosynthesis)	Sugar transferase	Experimental validation	Javed *et al.,* 2012
CJ1137c	Glycosyltransferase	Putative glycosyltransferase (LOS biosynthesis)	Sugar transferase	Conservation	Gundogdu *et al*., 2007
CJ1138	Glycosyltransferase	Putative glycosyltransferase (LOS biosynthesis)	Sugar transferase	Conservation	Parkhill *et al*., 2000
CJ1139c	*wlaN;* beta-1,3 galactosyltransferase	*wlaN;* beta-1,3 galactosyltransferase (LOS biosynthesis)	Sugar transferase	Experimental validation	Linton *et al.,* 2000
CJ1142*	*neuC1;* UDP-N-acetylglucosamine 2-epimerase	Epimerase, putative bifunctional glycosyltransferase (LOS biosynthesis)	UDP-Glycosyltransferase	-	-
CJ1143*	*neuA1;* bifunctional beta-1,4-N-acetylgalactosaminyltransferse/CMP-Neu5Ac synthase	Bifunctional beta-1,4-N-acetylgalactosaminyltransferse/CMP-Neu5Ac synthase, putative glycosyltransferase (LOS biosynthesis)	Sugar transferase	-	-
CJ1145c*	Hypothetical protein	Putative glycosyltransferase (LOS biosynthesis)	Sugar transferase	-	-
CJ1146c	*waaV;* glycosyltransferase	*waaV;* putative glycosyltransferase (LOS biosynthesis)	Sugar transferase	Conservation	Gundogdu *et al*., 2007
CJ1148	*waaF;* heptosyltransferase II	*waaF;* heptosyltransferase II (LOS biosynthesis)	UDP-Glycosyltransferase	Experimental validation	Oldfield *et al.,* 2002
CJ1311*	*pseF;* acylneuraminate cytidylyltransferase	Acylneuraminate cytidylyltransferase, putative glycosyltransferase (Protein O-glycosylation)	Sugar transferase	-	-
CJ1312[#]	*pseG;* nucleotidase	*pseG;* nucleotidase	UDP-Glycosyltransferase	Experimental validation (of other than the GT activity)	Liu & Tanner, 2006

CJ1328*	*neuC2;* UDP-N-acetylglucosamine 2-epimerase	Epimerase, putative bifunctional glycosyltransferase (Protein O-glycosylation)	UDP-Glycosyltransferase	-	-
CJ1329*	Sugar-phosphate nucleotide transferase	Putative glycoysltransferase (Protein O-glycosylation)	Sugar transferase	-	-
CJ1331*	*ptmB;* acylneuraminate cytidylyltransferase	Putative glycosyltransferase (Protein O-glycosylation)	Sugar transferase	-	-
CJ1333*	*pseD;* PseD protein	Putative glycosyltransferase (Protein O-glycosylation)	Sugar transferase	-	-
CJ1349c*	Fibronectin/fibrinogen-binding protein	Putative glycosyltransferase	Pfam/CAZy	-	-
CJ1350*	*mobA;* molybdopterin-guanine dinucleotide biosynthesis protein	Putative glycoysltransferase	Sugar transferase	-	-
CJ1416c*	Sugar nucleotidyltransferase	Putative glycoysltransferase	Sugar transferase	-	-
CJ1423c[#]	*hddC;* D-glycero-D-manno-heptose 1-phosphate guanosyltransferase	*hddC;* D-glycero-D-manno-heptose 1-phosphate guanosyltransferase	Sugar transferase	Conservation	Valvano *et al.,* 2002; Gundogdu *et al.,* 2007
CJ1432c	Sugar transferase	Putative glycosyltransferase (CPS biosynthesis)	UDP-Glycosyltransferase	Conservation	Gundogdu *et al*., 2007
CJ1434c	Sugar transferase	Putative glycosyltransferase (CPS biosynthesis)	Sugar transferase	Conservation	Gundogdu *et al*., 2007
CJ1438c	Sugar transferase	Putative glycosyltransferase (CPS biosynthesis)	Sugar transferase	Conservation	Gundogdu *et al*., 2007
CJ1440c	Sugar transferase	Putative glycosyltransferase (CPS biosynthesis)	Sugar transferase	Conservation	Gundogdu *et al*., 2007
CJ1442c	Sugar transferase	Putative glycosyltransferase (CPS biosynthesis)	Sugar transferase	Conservation	Gundogdu *et al*., 2007
CJ1536c*	*galU;* UTP-glucose-1-phosphate uridylyltransferase	Putative glycosyltransferase	Sugar transferase	-	-
CJ1607*	*ispDF;* bifunctional 2-C-methyl-D-erythritol 4-phsophate cytidylyltransferase/2-C-methyl-D-erythritol 2,4-cyclodiphosphate synthase	Bifunctional 2-C-methyl-D-erythritol 4-phsophate cytidylyltransferase/2-C-methyl-D-erythritol 2,4-cyclodiphosphate synthase, putative glycosyltransferase	Sugar transferase	-	-
CJ1610*	*pgpA;* putative phosphatidylglycerophosphatase	Putative glycosyltransferase	Pfam/CAZy	-	-
CJ1679*	Hypothetical protein	Putative glycoysltransferase	Pfam/CAZy	-	-
