# Supplementary material for: A network-based approach to identify substrate classes of bacterial glycosyltransferases
Source: BMC Genomics. 2014 May 8;15(1):349. doi: 10.1186/1471-2164-15-349 (PMC4039749; doi:10.1186/1471-2164-15-349)
Supplement: Supplementary file 2 — Additional file 2: Table S2: Proposed substrate classes of glycosyltransferases in Campylobacter jejuni NCTC 11168. Locus tag: gene identifier of the predicted GT used as query in STRING to obtain a query-dependent subnetwork. Localization: indicates whether the query-GT was predicted to be cytoplasmic (C) or a transmembrane protein (TM). Enriched GO categories: GO categories enriched amongst the members of the query-dependent subnetwork of the indicated query-GT. Only categories showing an enrichment value p < 0.05 are shown (according to a hypergeometric test corrected for multiple testing using False Discovery Rate). Membrane association: it refers to edges between the query-GT and members of its subnetwork predicted to be transmembrane proteins. Partner GTs: predicted/experimentally validated GTs that belong to the subnetwork of the query-GT. Predicted substrate class of a query-GT: inferred from the GO enrichment analysis of the query-dependent subnetwork of the indicated query-GT derived from STRING. Potential protein substrate: it refers to edges between the query-GT and members of its subnetwork predicted to have N- or O-glycosylation signals. Such proteins are therefore suggested to be potential substrates of the query-GT in the cases where proteins are the proposed substrate. Evidence: level of evidence for the substrate class prediction. Conservation: shows a significant sequence conservation with a GT for which a susbtrate specificity has been experimentally validated in a closely related species. Experimental validation: the substrate specificity of the GT has been experimentally validated in Campylobacter jejuni NCTC 11168. Reference: publication(s) supporting the predicted substrate class of the query-GT. (PDF 131 KB) [file 12864_2013_6075_MOESM2_ESM.pdf]

Table S2. Proposed substrate classes of glycosyltransferases in *Campylobacter jejuni* NCTC 11168. Locus tag: gene identifier of the predicted GT used as query in STRING to obtain a query-dependent subnetwork. **Localization**: indicates whether the query-GT was predicted to be cytoplasmic (C) or a transmembrane protein (TM). **Enriched GO categories**: GO categories enriched amongst the members of the query-dependent subnetwork of the indicated query-GT. Only categories showing an enrichment value p<0.05 are shown (according to a hypergeometric test corrected for multiple testing using False Discovery Rate). **Membrane association**: it refers to edges between the query-GT and members of its subnetwork predicted to be transmembrane proteins. **Partner GTs**: predicted/experimentally validated GTs that belong to the subnetwork of the query-GT. **Predicted substrate class of a query-GT**: inferred from the GO enrichment analysis of the query-dependent subnetwork of the indicated query-GT derived from STRING. **Potential protein substrate**: it refers to edges between the query-GT and members of its subnetwork predicted to have *N*- or *O*-glycosylation signals. Such proteins are therefore suggested to be potential substrates of the query-GT in the cases where proteins are the proposed substrate. **Evidence**: level of evidence for the substrate class prediction. **Conservation**: shows a significant sequence conservation with a GT for which a susbtrate specificity has been experimentally validated in a closely related species. Experimental validation: the substrate specificity of the GT has been experimentally validated in *Campylobacter jejuni* NCTC 11168. **Reference**: publication(s) supporting the predicted substrate class of the query-GT.

Query-GT locus tag	Query-GT localization	Enriched GO categories	Membrane association	Partner GTs	Predicted substrate class	Potential protein substrate	Evidence	Reference
CJ1133	C	CPS biosynthetic process	CJ1134 (lipid A biosynthesis lauroyl acyltransferase)	CJ1135; CJ1148; CJ1127c	CPS	-	Experimental validation	Kanipes *et al.*, 2006
CJ1416c	C	Polysaccharide biosynthetic process; Polysaccharide transport	-	-	CPS	-	Conservation	Gundogdu *et al.*, 2007
CJ1423c	C	CPS biosynthetic process	-	-	CPS	-	Conservation	Gundogdu *et al.*, 2007
CJ1432c	C	CPS biosynthetic process	-	CJ1434c	CPS	-	Conservation	Gundogdu *et al.*, 2007
CJ1434c	C	Lipopolysaccharide biosynthetic process	-	CJ1432c; CJ1136; Cj1434c; Cj1440c	CPS	-	Conservation	Gundogdu *et al.*, 2007
CJ1438c	C	CPS biosynthetic process	CJ1440c (Putative sugar transferase)	Cj1125c; CJ1440c	CPS	-	Conservation	Gundogdu *et al.*, 2007
CJ1440c	TM	Polysaccharide biosynthetic process; Polysaccharide transport		CJ1434c; CJ1438c; CJ1442c	CPS	-	Conservation	Gundogdu *et al.*, 2007
CJ1442c	C	Polysaccharide transport	CJ1440c (Putative sugar transferase)	CJ1440c	CPS	-	Conservation	Gundogdu *et al.*, 2007

CJ1133	C	Lipopolysaccharide biosynthetic process	CJ1134 (lipid A biosynthesis lauroyl acyltransferase)	CJ1135; CJ1148; CJ1127c	LOS	-	Experimental validation	Kanipes *et al.*, 2006
CJ1135	C	Lipopolysaccharide biosynthetic process	CJ1134 (lipid A biosynthesis lauroyl acyltransferase)	CJ1148; CJ1133; CJ1136; CJ1127c	LOS	-	Conservation	Taboada *et al*., 2007
CJ1136	C	Lipopolysaccharide biosynthetic process	-	CJ1135; CJ1133; CJ1124c; CJ1128c; CJ1146c; CJ1129c; CJ1434c	LOS	-	Experimental validation	Javed *et al.*, 2012
CJ1137c	C	Lipopolysaccharide biosynthetic process; Transcription termination	-	CJ1145c; CJ1138; CJ1143; CJ1139c	LOS	-	Conservation	Gundogdu *et al.*, 2007
CJ1138	C	Lipopolysaccharide biosynthetic process	-	CJ1148; CJ1124c; CJ1137c; CJ1125c; CJ1146c; CJ1133; CJ1434c	LOS	-	Conservation	Gundogdu *et al.*, 2007
CJ1139c	C	Lipopolysaccharide biosynthetic process; N-acetylneuraminate biosynthetic process	-	CJ1148; CJ1124c; CJ1125c; CJ1142; CJ1143; CJ1127c	LOS	-	Experimental validation	Linton *et al.*, 2000
CJ1142	C	Lipopolysaccharide biosynthetic process; N-acetylneuraminate biosynthetic process	-	CJ1331; CJ1124c; CJ1143; CJ1139c	LOS	-	Conservation	Gundogdu *et al.*, 2007
CJ1143	C	Lipopolysaccharide biosynthetic process; N-acetylneuraminate biosynthetic process	-	CJ1124c; CJ1137c; CJ1328; CJ1142; CJ1139c	LOS	-	Conservation	Gundogdu *et al.*, 2007
CJ1145c	C	Lipopolysaccharide biosynthetic process	-	CJ1442c; CJ1138; CJ1137c; CJ1146c; CJ1136;	LOS	-	Conservation	Gundogdu *et al.*, 2007

				CJ1133; CJ1139c				
CJ1146c	C	Lipopolysaccharide biosynthetic process	-	CJ1148; CJ1124c; CJ1128c; CJ1133; CJ1127c; CJ1145c	LOS	-	Conservation	Gundogdu *et al.,* 2007
CJ1148	C	Lipopolysaccharide biosynthetic process	CJ1134 (lipid A biosynthesis lauroyl acyltransferase)	CJ1135; CJ1146c; CJ1133; CJ1127c	LOS	-	Experimental validation	Oldfield *et al.*, 2002
CJ0508	TM	Peptidoglycan-based cell wall biogenesis	-	CJ1039	PG	-	Conservation	Murray *et al.,* 1997
CJ1039	C	Peptidoglycan-based cell wall biogenesis; Regulation of cell shape; Cell cycle	CJ1038 (putative cell division/peptidoglycan biosynthesis protein)	CJ0508	PG	-	Conservation	Mengin-lecreulx *et al.,* 1991
CJ1124c	TM	Galactose metabolic process; Amino acid modification	-	CJ1125c; CJ1128c; CJ1121c; CJ1129c; CJ1127c	Protein N-glycosylation	-	Experimental validation	Glover *et al.,* 2006
CJ1125c	C	Galactose metabolic process; Protein amino acid glycosylation	CJ1124c (Galactosyltransferase)	CJ1126c; CJ1124c; CJ1128c; CJ1127c; CJ1129c;	Protein N-glcyosylation	-	Experimental validation	Glover *et al.* PNAS, 2005
CJ1126c	TM	Protein amino acid glycosylation	-	CJ1124c; CJ1128c; CJ1125c; CJ1129c; CJ1127c	Protein N-glycosylation	CJ0367c (*cme*A - periplasmic fusion protein CmeA)	Experimental validation	Glover *et al.,* Chem. Biol., 2005
CJ1127c	C	Galactose metabolic process; Protein amino acid glycosylation	CJ1126c (oligosaccharide transferase to N-glycosylate proteins)	CJ1126c; CJ1124c; CJ1128c; CJ1125c; CJ1129c	Protein N-glycosylation	-	Experimental validation	Glover *et al,* PNAS, 2005
CJ1128c	C	Galactose metabolic process; Protein amino acid glycosylation	CJ1126c (oligosaccharide transferase to N-glycosylate proteins)	CJ1126c; CJ1124c; CJ1125c; CJ1129c; CJ1127c	Protein N-glycosylation	-	Experimental validation	Glover *et al.,* PNAS, 2005
CJ1129c	C	Galactose metabolic process;	CJ1130c (*pgl*k - flippase)	CJ1126c; CJ1124c;	Protein N-glycosylation	-	Experimental validation	Glover *et al.,* PNAS, 2005

		Protein amino acid glycosylation		CJ1128c; CJ1125c; CJ1127c				
CJ1328	C	Nucleotide-sugar metabolic process	-	CJ1331; CJ1311; CJ1329	Protein O-glycosylation	-	Conservation	Gundogdu *et al.*, 2007
CJ1329	C	Nucleotide-sugar metabolic process	-	CJ1331; CJ1333; CJ1328	Protein O-glycosylation	-	Conservation	Gundogdu *et al.*, 2007
CJ1331	C	Nucleotide-sugar metabolic process	-	CJ1333; CJ1328; CJ1329	Protein O-glycosylation	-	Conservation	Gundogdu *et al.*, 2007
CJ1333	C	Nucleotide-sugar metabolic process	-	CJ1331; CJ1328; CJ1329	Protein O-glycosylation	-	Conservation	Gundogdu *et al.*, 2007
